# Association between pupillary examinations and prognosis in patients with out-of-hospital cardiac arrest who underwent extracorporeal cardiopulmonary resuscitation: a retrospective multicentre cohort study

**DOI:** 10.1186/s13613-024-01265-7

**Published:** 2024-03-06

**Authors:** Takuro Hamaguchi, Toru Takiguchi, Tomohisa Seki, Naoki Tominaga, Jun Nakata, Takeshi Yamamoto, Takashi Tagami, Akihiko Inoue, Toru Hifumi, Tetsuya Sakamoto, Yasuhiro Kuroda, Shoji Yokobori, the SAVE-J II study group

**Affiliations:** 1 Department of Emergency and Critical Care Medicine, Nippon Medical School, 1-1-5 Sendagi, Bunkyo-ku, Tokyo, Japan; 2 Department of Healthcare Information Management, The University of Tokyo Hospital, Tokyo, Japan; 3 Division of Cardiovascular Intensive Care, Department of Cardiovascular Medicine, Nippon Medical School Hospital, Tokyo, Japan; 4 Department of Clinical Epidemiology and Health Economics, School of Public Health, The University of Tokyo, Tokyo, Japan; 5 Department of Emergency and Critical Care Medicine, Hyogo Emergency Medical Center, Kobe, Japan; 6 Department of Emergency and Critical Care Medicine, St. Luke’s International Hospital, Tokyo, Japan; 7 Department of Emergency Medicine, Teikyo University School of Medicine, Tokyo, Japan; 8 Department of Emergency Medicine, Kagawa University School of Medicine, Kagawa, Japan

**Keywords:** Extracorporeal cardiopulmonary resuscitation, Favourable neurological outcome, Out-of-hospital cardiac arrest, Pupillary examination

## Abstract

**Background:**

In some cases of patients with out-of-hospital cardiac arrest (OHCA) who underwent extracorporeal cardiopulmonary resuscitation (ECPR), negative pupillary light reflex (PLR) and mydriasis upon hospital arrival serve as common early indicator of poor prognosis. However, in certain patients with poor prognoses inferred by pupil findings upon hospital arrival, pupillary findings improve before and after the establishment of ECPR. The association between these changes in pupillary findings and prognosis remains unclear. This study aimed to clarify the association of pupillary examinations before and after the establishment of ECPR in patients with OHCA showing poor pupillary findings upon hospital arrival with their outcomes. To this end, we analysed retrospective multicentre registry data involving 36 institutions in Japan, including all adult patients with OHCA who underwent ECPR between January 2013 and December 2018. We selected patients with poor prognosis inferred by pupillary examinations, negative pupillary light reflex (PLR) and pupil mydriasis, upon hospital arrival. The primary outcome was favourable neurological outcome, defined as Cerebral Performance Category 1 or 2 at hospital discharge. Multivariable logistic regression analysis was performed to evaluate the association between favourable neurological outcome and pupillary examination after establishing ECPR.

**Results:**

Out of the 2,157 patients enrolled in the SAVE-J II study, 723 were analysed. Among the patients analysed, 74 (10.2%) demonstrated favourable neurological outcome at hospital discharge. Multivariable analysis revealed that a positive PLR at ICU admission (odds ration [OR] = 11.3, 95% confidence intervals [CI] = 5.17–24.7) was significantly associated with favourable neurological outcome. However, normal pupil diameter at ICU admission (OR = 1.10, 95%CI = 0.52–2.32) was not significantly associated with favourable neurological outcome.

**Conclusion:**

Among the patients with OHCA who underwent ECPR and showed poor pupillary examination findings upon hospital arrival, 10.2% had favourable neurological outcome at hospital discharge. A positive PLR after the establishment of ECPR was significantly associated with favourable neurological outcome.

## Background

Current guidelines recommend delaying the evaluation of neurological prognosis in post-conventional cardiopulmonary resuscitation (CCPR) and extracorporeal cardiopulmonary resuscitation (ECPR) patients with out-of-hospital cardiac arrest (OHCA) by 72 h [1–4]. Early prognostication is difficult because of persistently reduced consciousness and haemodynamic instability in severely ill cardiac arrest survivors [5]. However, 30–50% of ECPR patients have reported experiencing early withdrawal of life-sustaining therapy within 72 h, mainly because of the perceived poor neurological prognosis [6, 7]. Errors in prognostication can have significant consequences. For example, excessive prognostic pessimism may lead to inappropriate treatment withdrawal [8]. Therefore, it is crucial to accurately evaluate and avoid misjudgement in the early phase of neurological prognosis in patients expected to have a poor prognosis.

A previous study suggested that pupillary examination is one of the common predictors of early neurological prognosis in ECPR patients [2]. Specifically, a negative pupillary light reflex (PLR) and mydriasis upon hospital arrival are associated with poor prognostic outcomes [9, 10]. However, several previous studies on pupil examinations upon hospital arrival have shown that even if negative PLR and mydriasis are observed, their sensitivity regarding neurological prognosis is low and insufficient to predict poor neurological prognosis, which is an important decision [11, 12]. On the other hands, it has occasionally been observed that pupillary findings change after the return of spontaneous circulation (ROSC) or extracorporeal membrane oxygenation (ECMO) establishment in patients with OHCA in whom poor prognosis was inferred from pupillary findings at the time of initial evaluation [13, 14]. Despite the potential relevance to prognosis, the association between these changes in pupillary examination and the neurological prognosis in patients undergoing ECPR has not been well established.

This study aims to clarify the association between pupillary examinations before and after ECMO establishment and the prognosis at hospital discharge in ECPR patients with pupillary examination findings inferring poor prognosis, who had negative pupillary light reflex and mydriasis pupil upon hospital arrival.

## Methods

### Data source

This was a retrospective observational study that utilised the SAVE-J II database, a multicentre registry study on ECPR for patients with OHCA involving 36 participating institutions in Japan. The following data were retrieved from the database: patient characteristics, prehospital information, patient findings upon hospital arrival, diagnosis and intervention, mechanical support information, time course, body temperature management, intensive care unit (ICU) information, and outcomes. According to this database, most of the registered cases involve patients with OHCA who experience events near participating institutions where ECPR could be performed, and ECMO was introduced after being transferred to the hospital [15]. As indicated by previous study in our SAVE-J2 study group [16], inclusion criteria for ECPR in patients with OHCA were established in approximately 60% of the participant institutions. Approximately 40% of these institutions had no age limitations, and half of the institutions had exclusion criteria. None of the institutions had exclusion criteria for obesity, cachexia, or initial rhythm pulseless electrical activity. The absence of witnesses or bystander CPR were exclusions criteria in 10% of the institutions. Further details regarding characteristics of the participating institutions, initial resuscitation management, ECMO initiation, ECMO management, intra-aortic balloon pumping, endotracheal intubation, management during coronary angiography, and CT criteria were described in a previous study.

### Study population

The SAVE-J II included adult patients (≥ 18 years of age) with OHCA who were admitted to the emergency department between January 1, 2013, and December 31, 2018, who had received ECPR. ECPR was defined as resuscitation from cardiac arrest using veno-arterial extracorporeal membrane oxygenation (VA-ECMO) [17]. In our secondary analysis, we included patients from the SAVE-J II who met the inclusion criteria of initiation of VA-ECMO before ICU admission, and excluded those who achieved ROSC upon hospital arrival and at ECMO initiation. In addition, patients with non-cardiac conditions and PLR-positivity or normal pupil diameter at the time of hospital admission were also excluded from the study. Noncardiac conditions included acute aortic dissection/aortic aneurysm, hypothermia, primary cerebral disorder, infections, drug intoxication, trauma, suffocation, and drowning [18]. Hypothermia was diagnosed by a physician or defined as a body temperature of less than 30℃ at admission [18]. Furthermore, based on pupillary examination, we excluded patients with at least one instance of positive PLR or normal pupil diameter upon hospital arrival. In addition, patients with unknown outcomes in terms of prognosis and pupillary examination were excluded.

### Data collection and variable definition

The following patient data retrieved from the SAVE-J II database were used for the secondary analysis: age, sex, initial cardiac rhythm at the scene, witnessed cardiac arrest, bystander cardiopulmonary resuscitation (CPR), prehospital use of adrenaline and defibrillation, cause of cardiac arrest, percutaneous coronary intervention (PCI), time course, pupil diameter upon hospital arrival and ICU admission, PLR at hospital arrival and ICU admission, drug administration at ICU admission, length of ICU stay in days, length of hospital stay in days, and outcomes. Available patient’s data on drugs administrated at ICU admission included vasopressors, sedatives, and analgesics. Estimated low-flow time was defined as the time that elapsed between cardiac arrest and the establishment of ECMO if the cardiac arrest occurred in the ambulance or the time span between ambulance call and establishment of ECMO if cardiac arrest occurred in a location different from the ambulance. Time from hospital arrival to ICU admission was defined as that elapsed between hospital arrival and admission to ICU. Pupil diameters were recorded in the database in increments of 0.5 mm. Normal pupil diameter was defined as less than 4 mm, and mydriasis was defined as 4 mm or greater [19]. The contralateral light reflex was observed for the presence or absence of pupillary constriction; PLR positive was defined as pupillary constriction in response to light, and PLR negative was defined as the absence of pupillary constriction in response to light.

### Outcomes

The primary outcome was a favourable neurological outcome. Assessment of neurological outcomes at the time of hospital discharge was based on the cerebral performance category (CPC) score [20]. The CPC scores were defined as follows: CPC 1, conscious and alert with good cerebral performance; CPC 2, conscious and alert with moderate cerebral performance; CPC 3, conscious with severe cerebral disability; CPC 4, comatose or in a persistent vegetative state; and CPC 5, brain death or death. Neurological outcomes were dichotomised as favourable (CPC 1 or 2) or unfavourable (CPC 3–5) [20].

### Statistical analysis

Continuous variables are presented as means with standard deviations, while categorical variables are presented as counts with percentages. Baseline characteristics and pupillary examination findings according to favourable and unfavourable neurological outcomes were compared using Student’s t-test for continuous variables and Chi-square test for categorical variables. Thereafter, patients were categorised based on the pupillary examination findings at the time of ICU admission. The association between the outcomes and pupillary examination findings was evaluated using univariable and multivariable logistic regression analyses. Multivariable logistic regression analyses, fitted with generalised estimating equations were performed to account for clustering within institutions. Adjustments were made according to selected covariates (age, sex, witnessed cardiac arrest, bystander CPR, shockable rhythm at the scene, estimated low-flow time, prehospital epinephrine administration, positive PLR at ICU admission, normal pupil diameter at ICU admission, vasopressors administration at ICU admission, sedatives administration at ICU admission, and analgesics administration at ICU admission) based on previous studies [21]. Data were reported as odds ratios (OR) with 95% confidence intervals (CI). All statistical analyses were performed using the R software (version 4.2.2; R Foundation for Statistical Computing, Vienna, Austria). Two-sided values of *P* < 0.05 were considered statistically significant.

## Results

We enrolled 2,157 adult patients with OHCA who underwent ECPR between 2013 and 2018, according to the SAVE-J II. Of these, nine patients were excluded due to the implementation of VA-ECMO after ICU admission, and one was excluded as the cannulation was withdrawn due to ROSC. A total of 342 patients were excluded from the study because they had OHCA due to non-cardiac conditions. Furthermore, 96 patients who achieved ROSC upon hospital arrival and 58 who achieved ROSC upon ECMO initiation were also excluded. Additionally, five patients transferred from another hospital were excluded.

**Fig. 1:**
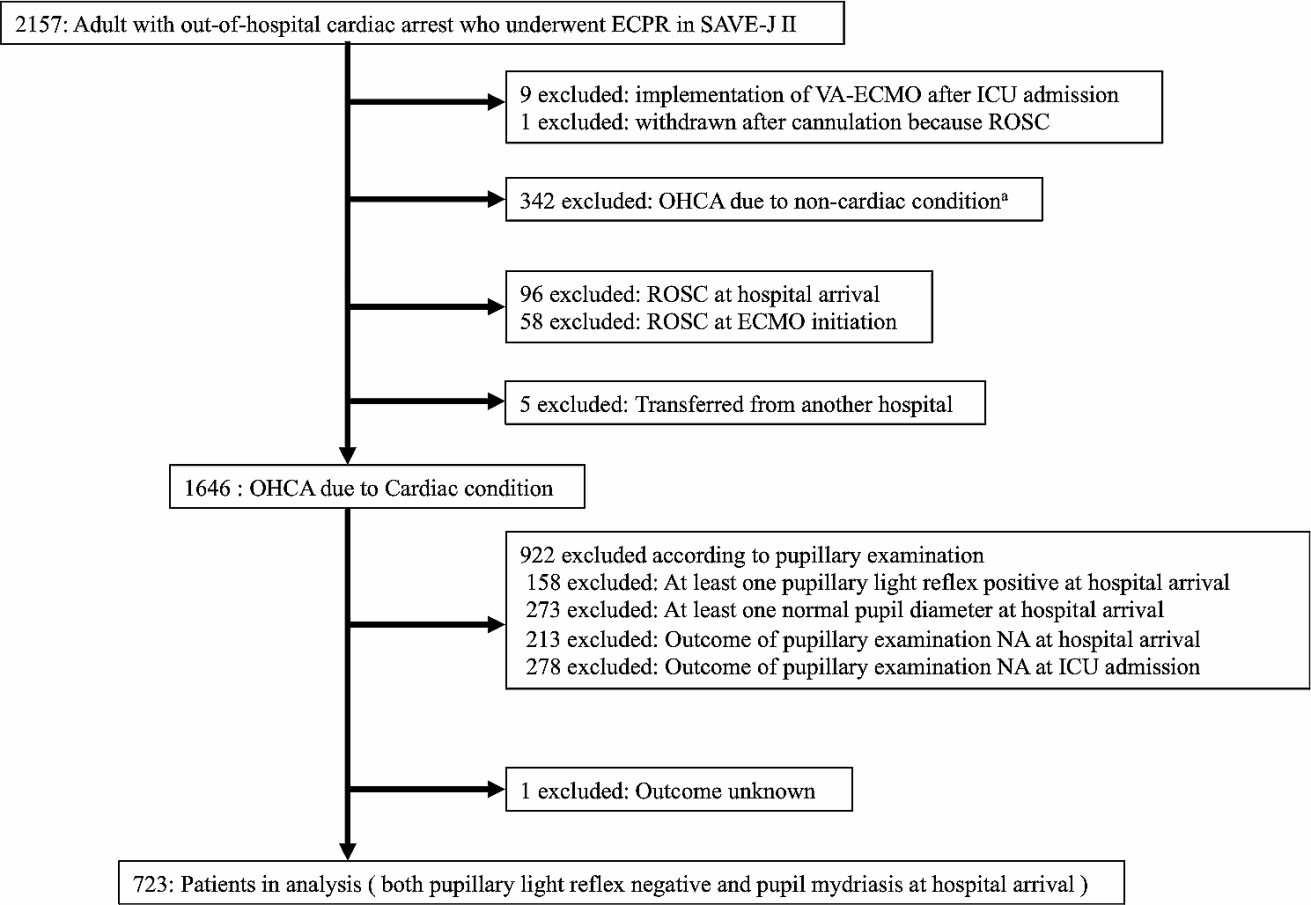
Flowchart of enrollment of study participants. ECPR: extracorporeal cardiopulmonary resuscitation; ECMO: extracorporeal membrane oxygenation; OHCA: out of hospital cardiac arrest; ROSC: return of spontaneous circulation. ^a^ non-cardiac condition: Acute aortic dissection / aortic aneurysm = 119, Hypothermia = 112, Primary cerebral disorders = 51, Infection = 20, Drug intoxication = 14, Trauma = 11, Suffocation = 8, Drowning = 4, Other external causes = 3

Among the 1,646 patients with OHCA due to cardiac conditions, 922 were excluded based on the pupillary examination findings. In addition, one patient was excluded as the outcome was unknown. Finally, 723 patients who presented with both PLR negativity and pupil mydriasis upon hospital arrival were analysed (Fig. 1).

Table 1 shows the comparison of baseline characteristics according to favourable neurological outcomes at the time of hospital discharge. A total of 74 (10.2%) patients comprised the favourable neurological outcome group. The favourable neurological outcome group was significantly younger, had a lower male proportion, higher proportion of shockable rhythm on arrest, a higher proportion of witnessed cardiac arrests, higher proportion of bystander CPR, lower proportion of prehospital epinephrine administration, higher proportion sedatives and analgesics administration at ICU admission, shorter estimated low-flow time and a longer length of ICU and hospital stay days compared with the unfavourable neurological outcome group.

**Table 1:** Baseline characteristics ^a^

	Favourable neurological outcome at hospital discharge *n* = 74		Unfavourable neurological outcome at hospital discharge *n* = 649		*P* value
Age, years	54.3	(14.7)	58	(13.1)	0.032
Sex, male	53.0	(71.6)	553	(85.2)	0.005
Initial rhythm at the scene					0.016
Shockable rhythm	65	(87.8)	463	(71.9)	
Pulseless electric activity	7	(9.5)	142	(21.9)	
Asystole	1	(1.4)	39	(6.0)	
Witnessed cardiac arrest	64	(86.5)	504	(77.7)	0.002
Bystander CPR	49	(66.2)	377	(58.1)	0.026
Prehospital intervention					
Epinephrine administration	14	(18.9)	240	(37.0)	0.009
Defibrillation	55	(74.3)	427	(65.8)	0.176
Cardiogenic cardiac arrest	65	(87.8)	560	(86.3)	0.817
Percutaneous coronary intervention	39	(52.7)	315	(48.5)	0.467
Drug administration at ICU admission^b^					
Vasopressors^c^	23	(31.1)	291	(44.8)	0.051
Sedatives^d^	55	(74.3)	316	(48.7)	< 0.001
Analgesics^e^	55	(74.3)	289	(44.5)	< 0.001
Body temperature at ICU admission, ℃	34.4	(1.4)	34.4	(1.2)	0.957
Estimated low flow time^f^, minutes	49.9	(13.7)	55.4	(18.5)	0.016
Time from hospital arrival to ICU admission^g^, minutes	197.4	(77.1)	199.2	(89.0)	0.871
Length of ICU stay, in days	14.0	(9.7)	6.7	(9.1)	< 0.001
Length of hospital stay^j^, in days	44.0	(34.0)	12.2	(21.7)	< 0.001

^a^ Data are presented as mean (SD) for continuous variables and as N (percentage) for categorical variables

CPR: cardiopulmonary resuscitation; ECMO: extracorporeal membrane oxygenation; ICU: intensive care unit

^b^ Drug administration at ICU admission refers to drugs administered to patients upon their admission to the ICU

^c^ Vasopressors include noradrenaline, dobutamine, dopamine, and adrenaline

^d^ Sedatives include propofol, midazolam, dexmedetomidine

^e^ Analgesics include fentanyl and morphine

^f^ Estimated low flow time is defined as the time from cardiac arrest to the establishment of ECMO if the location of cardiac arrest is ambulance and the time from calling an ambulance to the establishment of ECMO if the location of cardiac arrest is other than ambulance

^g^ Time from hospital arrival to ICU admission is time from time from hospital arrival to admission to ICU

^h^ Length of hospital stay includes both intensive care unit and hospital days

Missing data: Age = 0, Gender = 0, Initial rhythm at the scene = 0, Witnessed cardiac arrest = 1, Bystander CPR = 5, Epinephrine administration = 7, Defibrillation = 4, Cardiac arrest caused by cardiogenic = 41, Percutaneous coronary intervention = 18, Vasopressors = 22, Sedatives = 20, Analgesics = 11, Body temperature at ICU admission = 114, Estimated low flow time = 24, Time from hospital arrival to ICU admission = 40, Length of ICU stay = 5, Length of hospital stay = 1

Table 2 shows the pupillary examination findings according to the neurological outcomes. Pupil diameters were significantly smaller upon hospital arrival and at the time of ICU admission in the favourable neurological outcome group than in the unfavourable neurological outcome group. There was a higher proportion of PLR positivity at the time of ICU admission in the favourable neurological outcome group than in the unfavourable neurological outcome group (81.1% vs. 29.4%, *P* < 0.001).

**Table 2:** Pupillary examination according to neurological outcome at hospital discharge ^a^

	Favourable neurological outcome at hospital discharge *n* = 74		Unfavourable neurological outcome at hospital discharge *n* = 649		*P* value
Left pupil diameter at hospital arrival, mm	5.0	(1.0)	5.5	(1.1)	< 0.001
Right pupil diameter at hospital arrival, mm	5.0	(1.1)	5.5	(1.1)	< 0.001
Left pupil diameter at ICU admission, mm	3.1	(1.1)	3.6	(1.7)	< 0.001
Right pupil diameter at ICU admission, mm	3.0	(1.1)	3.6	(1.7)	< 0.001
PLR positive at ICU admission	60	(81.1)	191	(29.4)	< 0.001

^a^ Data are presented as mean (SD) for continuous variables and as N (percentage) for categorical variables

ICU, intensive care unit; PLR, pupillary light reflex

Missing data: Left pupil diameter at hospital arrival = 0, Right pupil diameter at hospital arrival = 0, Left pupil diameter at ICU admission = 0, Right pupil diameter at ICU admission = 0, PLR positive at ICU admission = 0

Table 3 shows the baseline characteristics and prognoses classified by pupillary examination at the time of ICU admission. Patients were classified into four groups: 253 patients in the PLR-negative group with pupil mydriasis, 219 in the PLR-negative group with normal pupil diameter, 45 in the PLR-positive group with pupil mydriasis and 206 in the PLR-positive group with normal pupil diameter. A total of 4 (1.6%), 10 (4.6%), 13 (28.9%), and 47 (22.8%) patients in the abovementioned four groups had favourable neurological outcomes at the time of hospital discharge; and 11 (4.3%), 51 (23.3%), 26 (57.8%), and 107 (51.9%) survived until discharge. Among patients with positive PLR at ICU admission, 23.9% had favourable neurological outcome, among patients with normal pupil diameter at ICU admission, 13.4% had favourable neurological outcomes.

**Table 3:** Baseline characteristics and prognosis classified by pupillary examination at ICU admission ^a^

	Overall population		Negative PLR^b^ and pupil mydriasis^d^		Negative PLR^b^ and normal pupil diameter^e^		Positive PLR^c^ and pupil mydriasis^d^		Positive PLR^c^ and normal pupil diameter^e^	
	*n* = 723		*n* = 253		*n* = 219		*n* = 45		*n* = 206	
Age, years	57.5	(13.3)	56.4	(13.2)	59.4	(12.8)	53.27	(13.1)	57.6	(13.7)
Sex, male	606	(83.8)	219	(86.6)	189	(86.3)	37	(82.2)	161	(78.2)
Initial rhythm at the scene										
Shockable rhythm at the scene	528	(73.1)	175	(69.7)	161	(74.2)	39	(86.7)	153	(75.0)
Pulseless electric activity	149	(20.6)	56	(22.1)	45	(20.5)	6	(13.3)	42	(20.4)
Asystole	40	(5.5)	20	(7.9)	11	(5.0)	0	(0.0)	9	(4.4)
Witnessed cardiac arrest	568	(78.6)	197	(77.9)	171	(78.1)	40	(88.9)	160	(77.7)
Bystander CPR	426	(58.9)	148	(58.5)	128	(58.4)	31	(68.9)	119	(57.8)
Prehospital intervention										
Epinephrine administration	254	(35.1)	99	(39.1)	76	(34.7)	17	(37.8)	62	(30.1)
Defibrillation	482	(66.7)	152	(60.1)	154	(70.3)	35	(77.8)	141	(68.4)
Cardiogenic cardiac arrest	625	(86.4)	211	(83.4)	194	(88.6)	40	(88.9)	180	(87.4)
Percutaneous coronary intervention	354	(49.0)	109	(43.1)	105	(47.9)	22	(48.9)	118	(57.3)
Drug administration at ICU admission^f^										
Vasopressors^g^	314	(43.4)	128	(53.3)	94	(44.3)	19	(43.2)	73	(35.6)
Sedatives^h^	371	(51.3)	79	(32.6)	136	(63.8)	25	(56.8)	131	(64.2)
Analgesics^i^	344	(47.6)	91	(36.3)	114	(53.3)	25	(56.8)	114	(56.2)
Body temperature at ICU admission, ℃	34.4	(1.2)	34.4	(1.4)	34.4	(1.1)	34.8	(1.2)	34.2	(1.2)
Estimated low flow time^j^	54.8	(18.1)	59.7	(18.0)	52.4	(19.2)	55.73	(20.8)	51.4	(15.0)
Time from hospital arrival to ICU admission^k^	199.0	(87.7)	188.2	(86.6)	200.9	(95.9)	192.71	(56.0)	211.7	(84.7)
Favourable neurological outcome at hospital discharge	74	(10.2)	4	(1.6)	10	(4.6)	13	(28.9)	47	(22.8)
Survival to hospital discharge	195	(27.0)	11	(4.3)	51	(23.3)	26	(57.8)	107	(51.9)
CPC score at hospital discharge										
1	59	(8.2)	3	(1.2)	8	(3.7)	10	(22.2)	38	(18.4)
2	15	(2.1)	1	(0.4)	2	(0.9)	3	(6.7)	9	(4.4)
3	28	(3.9)	1	(0.4)	7	(3.2)	5	(11.1)	15	(7.3)
4	93	(12.9)	6	(2.4)	34	(15.5)	8	(17.8)	45	(21.8)
5	528	(73.0)	242	(95.7)	168	(76.7)	19	(42.2)	99	(48.1)
Length of ICU stay, days	7.4	(9.4)	3.1	(3.8)	7.8	(9.2)	14.1	(12.7)	10.9	(11.2)
Length of hospital stay^l^, days	15.5	(25.2)	4.6	(14.8)	15.2	(23.0)	31.2	(32.1)	25.7	(29.5)

^a^ Data are presented as mean (SD) for continuous variables and as N (percentage) for categorical variables

PLR: pupillary light reflex; ECMO: extracorporeal membrane oxygenation; ICU: intensive care unit; CPC: cerebral performance category

^b^ At least one PLR is negative at ICU admission

^c^ Both PLR are positive at ICU admission

^d^ At least one pupil diameter is mydriasis at ICU admission

^e^ Both pupil diameters are normal at ICU admission

^f^ Drug administration at ICU admission refers to drugs administered to patients upon their admission to the ICU

^g^ Vasopressors include noradrenaline, dobutamine, dopamine, and adrenaline

^h^ Sedatives include propofol, midazolam, and dexmedetomidine

^i^ Analgesics include fentanyl and morphine

^j^ Estimated low flow time is defined as the time from cardiac arrest to the establishment of ECMO if the location of cardiac arrest is ambulance and the time from calling an ambulance to the establishment of ECMO if the location of cardiac arrest is other than ambulance

^k^ Time from hospital arrival to ICU admission is time from time from hospital arrival to admission to ICU

^l^ Length of hospital stay includes both intensive care unit and hospital days

Missing data: Age = 0, Gender = 0, Initial rhythm at the scene = 0, Witnessed cardiac arrest = 1, Bystander CPR = 5, Epinephrine administration = 7, Defibrillation = 4, Cardiac arrest caused by cardiogenic = 41, Percutaneous coronary intervention = 18, Vasopressors = 22, Sedatives = 20, Analgesics = 11, Body temperature at ICU admission = 114, Estimated low flow time = 24, Time from hospital arrival to ICU admission = 40, Favourable neurological outcome at hospital discharge = 0, Survival to hospital discharge = 0, CPC score at hospital discharge = 0, Length of ICU stay = 5, Length of hospital stay = 1

Table 4 shows univariable and multivariable logistic regression analyses for favourable neurological outcomes. Adjusted ORs were derived from logistic regression models for age, sex, witnessed cardiac arrest, bystander CPR, shockable rhythm on arrest, estimated low-flow time, prehospital epinephrine administration, positive PLR at ICU admission, normal pupil diameter at ICU admission, vasopressors administration at ICU admission, sedatives administration at ICU admission, analgesics administration at ICU admission, and clustering within institutions. Multivariable analysis revealed positive PLR at ICU admission (adjusted OR = 11.3; 95%CI = 5.17–24.7) was significantly associated with favourable neurological outcomes at hospital discharge. The area under the receiver operating characteristic curve of this multivariate model is 87.9% (95%CI, 84.3–91.5). In terms of the predictive value of the positive PLR for favourable neurological outcomes, the positive predictive value was 24%, the sensitivity was 81%, with a false positive rate of 29.4%, and a false negative rate of 18.9%. Concerning normal pupil diameter, the positive predictive value was 13%, and the sensitivity was 77%, with a false positive rate of 56.7%, and a false negative rate of 23.0%.

**Table 4:** Unadjusted and adjusted association with favourable neurological outcome at hospital discharge

	Univariate analysis				Multivariable analysis^a^		
	OR	(95%CI)	P value		OR	(95%CI)	P value
Positive PLR at ICU admission^b^	10.28	(5.77–19.55)	< 0.001		11.3	(5.17–24.7)	< 0.001
Normal pupil diameter at ICU admission^c^	2.56	(1.49–4.63)	0.001		1.10	(0.52–2.32)	0.80
Age	0.98	(0.96–1.0)	0.032		0.98	(0.96–0.99)	0.063
Sex, male	0.44	(0.26–0.77)	0.003		0.47	(0.26–0.87)	0.016
Shockable rhythm on arrest	3.18	(1.58–7.30)	0.002		2.95	(1.36–6.44)	0.006
Witnessed cardiac arrest	2.05	(1.0–4.50)	0.052		1.56	(0.74–3.30)	0.24
Bystander CPR	1.52	(0.91–2.60)	0.11		1.28	(0.87–1.88)	0.21
Estimated low flow time^d^	0.98	(0.96–1.0)	0.014		0.99	(0.97–1.01)	0.348
Prehospital epinephrine administration	0.40	(0.21–0.71)	0.003		0.54	(0.32–0.91)	0.021
Drug administration at ICU admission^e^							
Vasopressors^f^	0.57	(0.33–0.95)	0.036		0.59	(0.36–0.98)	0.042
Sedatives^g^	3.68	(2.09–6.87)	< 0.001		1.70	(0.98–2.95)	0.057
Analgesics^h^	3.50	(2.06–6.17)	< 0.001		1.83	(0.92–3.66)	0.084

OR: odds ratio; CI: confidence interval; CPR: cardiopulmonary resuscitation; PLR: pupillary light reflex; ICU: intensive care unit

^a^ Adjusted OR are derived from logistic regression models adjusted for age, gender, witnessed cardiac arrest, bystander CPR, shockable rhythm on arrest, estimated low flow time, prehospital epinephrine administration, positive PLR at ICU admission, normal pupil diameter at ICU admission, vasopressors administration at ICU admission, sedatives administration at ICU admission, analgesics administration at ICU admission and and clustering within institutions

^b^ Positive PLR at ICU admission refers to both pupils exhibiting a positive PLR at ICU admission

^c^ Normal pupil diameter at ICU admission indicates that both pupil diameters are of normal size at ICU admission

^d^ Estimated low flow time is defined as the time from cardiac arrest to the establishment of ECMO if the location of cardiac arrest is ambulance and the time from calling an ambulance to the establishment of ECMO if the location of cardiac arrest is other than ambulance

^e^ Drug administration at ICU admission pertains to drugs administered to patients upon their admission to the ICU

^f^ Vasopressors include noradrenaline, dobutamine, dopamine, and adrenaline

^g^ Sedatives include propofol, midazolam, and dexmedetomidine

^h^ Analgesics include fentanyl, and morphine

Missing data: Age = 0, Male = 0, Shockable rhythm on arrest = 0, Witnessed cardiac arrest = 1, Bystander CPR = 5, Estimated low flow time = 24, Prehospital epinephrine administration = 7, Positive PLR at ICU admission = 0, Normal pupil diameter at ICU admission = 0, Vasopressors = 22, Sedatives = 20, Analgesics = 11

## Discussion

In our study, 723 (43.9%) of the 1,646 patients who underwent ECPR due to cardiogenic cardiac arrest had negative PLR and pupillary mydriasis upon hospital arrival. Among these, 74 (10.2%) had good neurological outcomes. This study revealed a significant association between positive PLR and favourable neurological outcomes at the time of ICU admission after the establishment of ECMO in patients with negative PLR and pupil mydriasis upon hospital arrival.

To the best of our knowledge, this is the largest sample size and largest number of participating institutions in a study exploring the association between pupil examination and prognosis in patients undergoing ECPR [9, 10, 14, 22–25]. 　Furthermore, our study focused on cases with negative PLR and pupil mydriasis upon hospital arrival, which makes it more specific in terms of the selected parameters compared with previous studies.

In our study, an improvement in the PLR at the time of ICU admission after the establishment of ECMO was associated with better neurological outcomes. We observed changes in the pupillary examination findings at an interval of 188.2 to 211.7 min between hospital arrival and ICU admission. A previous study focusing on patients with OHCA treated with ECPR evaluated pupillary findings and prognosis after ECMO establishment and reported that the improvement of PLR was associated with prognosis [14]. In another study focusing on patients with OHCA treated with CCPR, they evaluated pupillary findings and prognosis at 6 ± 2 h after ROSC, and reported that the improvement of pupillary findings was associated with prognosis [26]. Pupillary findings in patients with post-cardiac arrest change within a few hours of ROSC or ECMO establishment. The evaluation of pupillary examinations shortly after stabilizing systemic dynamics with ECMO, in addition to considering the time of hospital arrival, as indicated in this study, may be advantageous. This is because factors contributing to prognosis can be inferred under such circumstances.

Recent studies in Japan have reported that a negative PLR and mydriasis upon hospital arrival serves as early prognostic predictors in patients with OHCA treated with ECPR, and the favourable neurological outcome in patients with negative PLR upon hospital arrival ranged between 14.1 and 15.4% [9, 10]. However, in our study we observed that the proportion of patients with good neurological outcome was 23.9%, including some patients with improved PLR after the establishment of ECMO. In comparison to previous studies in Japan, our evaluation of some patients with good neurological outcome might be attributed to the re-evaluation of pupillary examinations shortly after ECMO establishment, allowing for more accurate prognosis. Several previous studies have highlighted the disadvantage faced by some patients due to early treatment withdrawal resulting from an early determination of poor prognosis in patients with OHCA [6, 27, 28]. The risk of excessive prognostic pessimism, potentially resulting in inappropriate treatment withdrawal, may be mitigated by recognizing the association between improvement in PLR after ECMO establishment in patients initially presented with negative PLR and pupil mydriasis upon hospital arrival, thus indicating a favourable neurological prognosis as demonstrated in this study.

In patients with cardiac arrest, pupil constricting cells, such as in the brainstem or the retina, reperfusion influenced by the stable circulatory dynamics after the establishment of ECMO could result in some neuronal recovery with improvement in PLR [9, 13, 14, 22, 26, 29]. In contrast, improvement in mydriasis after the establishment of ECMO was not significantly associated with a good neurological prognosis in this study. The regulation mechanism of pupil size in patients after cardiac arrest is affected by not only brain stem but upper spinal cord which is not directly associated with neurological cognitive function [26, 30]. Therefore, our assessment of neurological prognosis based on pupil size may be complex and inaccurate.

This study had several limitations. First, this was an observational retrospective study with variations in the inclusion criteria at each participating institution. Second, the pupillary findings in this study were based on subjective evaluation by the observers and, therefore, may lack objectivity. The use of automated pupillometry might have limited subjectivity and encouraged objectivity in evaluating pupillary examinations. In our study, some institutions might have used automatic pupillometry, but specific details regarding the patients assessed using either automatic pupillometry or the penlight method at each institution were not provided. Additionally, information regarding whether the measurements were taken in a sufficiently dark environment or in daylight was also lacking. Third, it should be noted that the assessment of neurological prognosis through pupillary examination has inherent limitations. Our evaluations focused on determining whether improved pupillary findings before and after ECMO correlated with favourable neurological outcomes. It is crucial to exercise caution, as the absence of improvement in PLR at the time of ICU admission should not be hastily interpreted as early poor prognostication, given the various factors that can influence pupillary findings, such as sedatives and vasopressors. Moreover, the evaluation of neurological prognosis in patients with OHCA requires not a single prognostic factor but a multi-model approach that includes brain imaging studies, EEG, SSEP, biomarkers, myoclonus, and other brainstem reflex, particularly corneal reflex [2, 31]. Fourth, the course of treatment after ICU admission was not evaluated. Fifth, only the outcomes at the time of hospital discharge alone were evaluated, while long-term outcomes were not. Sixths, we did not analyze the withdrawal of life-sustaining therapy, a factor that may be relevant to the life prognosis of patients with OHCA.

## Conclusions

Among the OHCA patients who underwent ECPR and showed poor pupillary examination findings upon hospital arrival, 10.2% had a favourable neurological outcome at the time of hospital discharge. In these patients, a positive PLR at the time of ICU admission was significantly associated with favourable neurological outcomes. Prospective study should be conducted to further validate the findings of the present study.

## Data Availability

The datasets used and/or analysed during the current study are available from the corresponding author on reasonable requests.

## Abbreviations

ECPR: extracorporeal cardiopulmonary resuscitation
CPR: cardiopulmonary resuscitation
OHCA: out-of-hospital cardiac arrest
CCPR: conventional cardiopulmonary resuscitation
ROSC: return of spontaneous circulation
ECMO: extracorporeal membrane oxygenation
PLR: pupillary light reflex
CPC: cerebral performance category
OR: odds ratio
CI: confidence interval
SAVE-J II: Study of Advanced life support for Ventricular fibrillation with Extracorporeal circulation in Japan
VA-ECMO: venoarterial extracorporeal membrane oxygenation
